# Fused 1,2,3-Dithiazoles: Convenient Synthesis, Structural Characterization, and Electrochemical Properties

**DOI:** 10.3390/molecules21050596

**Published:** 2016-05-06

**Authors:** Lidia S. Konstantinova, Ilia V. Baranovsky, Irina G. Irtegova, Irina Y. Bagryanskaya, Leonid A. Shundrin, Andrey V. Zibarev, Oleg A. Rakitin

**Affiliations:** 1 N. D. Zelinsky Institute of Organic Chemistry, Russian Academy of Sciences, 119991 Moscow, Russia; konstantinova_ls@mail.ru (L.S.K.); ilay679@rambler.ru (I.V.B.); 2 Nanotechnology Education and Research Center, South Ural State University, 454080 Chelyabinsk, Russia; 3 N. N. Vorozhtsov Institute of Organic Chemistry, Siberian Branch, Russian Academy of Sciences, 630090 Novosibirsk, Russia; irteg@nioch.nsc.ru (I.G.I.); bagryan@nioch.nsc.ru (I.Y.B.); shundrin@nioch.nsc.ru (L.A.S.); zibarev@nioch.nsc.ru (A.V.Z.); 4 Department of Natural Sciences, National Research University–Novosibirsk State University, 630090 Novosibirsk, Russia; 5 Department of Chemistry, National Research University–Tomsk State University, 634050 Tomsk, Russia

**Keywords:** fused 1,2,3-dithiazoles, synthesis, sulfur monochloride, X-ray diffraction, cyclic voltammetry

## Abstract

A new general protocol for synthesis of fused 1,2,3-dithiazoles by the reaction of cyclic oximes with S_2_Cl_2_ and pyridine in acetonitrile has been developed. The target 1,2,3-dithiazoles fused with various carbocycles, such as indene, naphthalenone, cyclohexadienone, cyclopentadiene, and benzoannulene, were selectively obtained in low to high yields. In most cases, the hetero ring-closure was accompanied by chlorination of the carbocyclic moieties. With naphthalenone derivatives, a novel dithiazole rearrangement (**15**→**13**) featuring unexpected movement of the dithiazole ring from α- to β-position, with respect to keto group, was discovered. Molecular structure of 4-chloro-5*H*-naphtho[1,2-*d*][1,2,3]dithiazol-5-one **13** was confirmed by single-crystal X-ray diffraction. Electrochemical properties of **13** were studied by cyclic voltammetry and a complex behavior was observed, most likely including hydrodechlorination at a low potential.

## 1. Introduction

1,2,3-Dithiazoles, the five membered sulfur-nitrogen heterocycles, are promising for science and technology because of their biological activity, unusual chemical transformations and interesting physical properties [1,2,3]. In particular it has been shown that the 1,2,3-dithiazole scaffold can be effectively used in the design and synthesis of stable neutral and negatively charged radicals (*i.e.*, radical anions)—actual or potential building blocks for molecule-based conductive and/or magnetic functional materials [4,5,6,7]. One can imagine that continued exploration of the 1,2,3-dithiazole chemistry is guaranteed to yield new compounds of fundamental and/or applied significance.

Normally, monocyclic 1,2,3-dithiazoles are prepared from 4,5-dichloro-1,2,3-dithiazolium chloride (the Appel salt) as the key synthon [8,9]. Benzo-fused 1,2,3-dithiazolium chlorides (the Herz salts) can be easily prepared by the Herz reaction from aromatic amines and sulfur monochloride S_2_Cl_2_. Although this reaction has been known for about one hundred years, it is still used nowadays as well [10,11].

Other synthetic precursors of fused 1,2,3-dithiazoles are cyclic oximes used in reactions with S_2_Cl_2_ in the presence of organic bases such as *N*-ethyldiisopropylamine (Hünig’s base) and triisobutylamine. The disadvantage of this method is that no general procedure is established and in all cases arduous purification of products by column chromatography is required [12,13,14,15].

During our ongoing work with S_2_Cl_2_ we have found that the reaction conditions and nature of the organic base have a crucial role influencing the yields of the target sulfur-nitrogen heterocycles [16,17,18,19]. It was shown that many nitrogen organic bases, such as tertiary amines, for example 1,4-diazabicyclooctane (DABCO), interact with S_2_Cl_2_ forming ionic complexes in some cases [17]. However, to the best of our knowledge, possible interaction/complexation between S_2_Cl_2_ and such a strong nitrogen organic base such as pyridine has not been investigated.

In this paper we report a study of a reaction between cyclic oximes and S_2_Cl_2_/pyridine covering selective synthesis of fused 1,2,3-dithiazoles together with their structural characterization by single-crystal X-ray diffraction (XRD) and investigation of their electrochemical properties by cyclic voltammetry (CV).

## 2. Results and Discussion

### 2.1. Syntheses

**Scheme 1:**
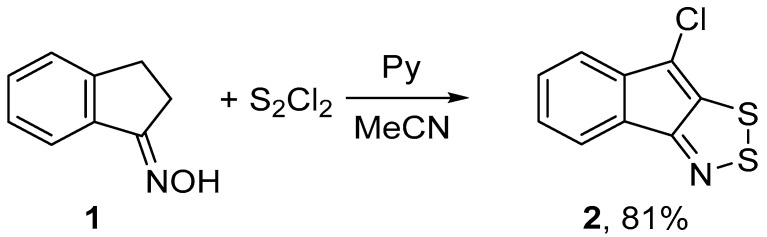
Reaction of oxime **1** with S_2_Cl_2_/pyridine to give dithiazole **2**.

In an effort to improve the synthesis of fused 1,2,3-dithiazoles, we re-investigated the reaction of 1-indanone oxime **1** with S_2_Cl_2_. Treatment of **1** with S_2_Cl_2_ in dimethylformamide (DMF), *i.e.*, a solvent which is frequently used in S_2_Cl_2_ reactions [16,19], in the temperature range from −25 to 20 °C gave 8-chloroindeno[1,2-*d*]-1,2,3-dithiazole **2** in low yields. Note, that in this case the hetero ring-closure was accompanied by chlorination. The type of base used was important for the success of reactions with S_2_Cl_2_ in other solvents, such as chloroform or acetonitrile (MeCN). Reaction of **1** with a two-fold excess of S_2_Cl_2_ and DABCO in chloroform at −5 °C led to complex mixtures containing **2** in the yield of 35%. The best results were achieved by treating **1** with a three-fold excess of S_2_Cl_2_ or a four-fold excess of pyridine in MeCN at 5 °C for 1 h which gave target **2** selectively in 81% yield (Scheme 1). The main feature of this and other S_2_Cl_2_/pyridine reactions is that the reaction mixtures are not tarry, and the product isolations do not require chromatography in contrast with the literature procedures [12,13,14,15].

**Scheme 2:**
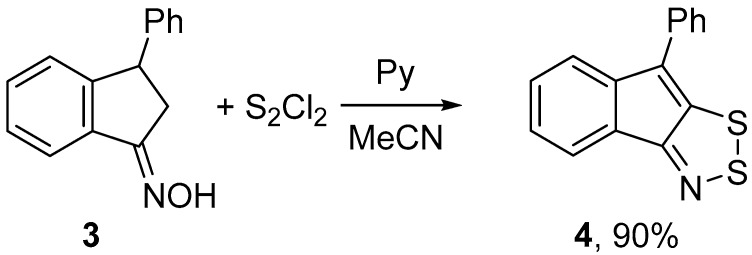
Reaction of oxime **3** with S_2_Cl_2_/pyridine to give dithiazole **4**.

Under the same conditions, 3-phenylindanone oxime **3** gave the corresponding 1,2,3-dithiazole **4** even in a higher yield, meanwhile chlorination was not observed (Scheme 2).

**Scheme 3:**
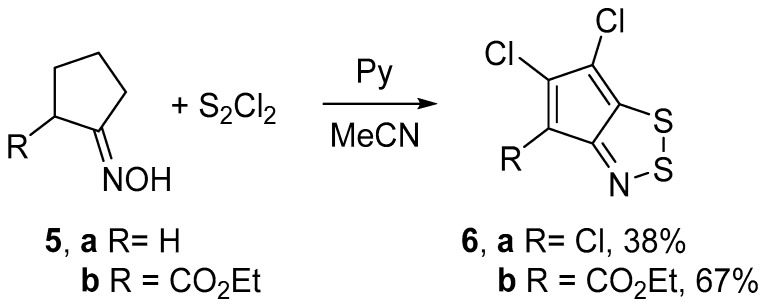
Reaction of oximes **5** with S_2_Cl_2_/pyridine to give dithiazoles **6**.

Treatment of the archetypal cyclopentanone oxime **5a** with an excess of S_2_Cl_2_ (6 equiv) and pyridine (8 equiv) in boiling MeCN gave 4,5,6-trichlorocyclopenta-1,2,3-dithiazole **6a** in moderate yield. Using lesser amounts of S_2_Cl_2_ or/and pyridine in an attempt to obtain less chlorinated product failed since only **6a** was isolated in lower yields. With a similar procedure, 4-carbethoxy substituted derivative **5b** was converted into dichlorocyclopentadithiazole **6b** in a good yield (Scheme 3). In both cases, the yields of dithiazoles **6** were slightly higher than those reported in the literature [12,15].

**Scheme 4:**
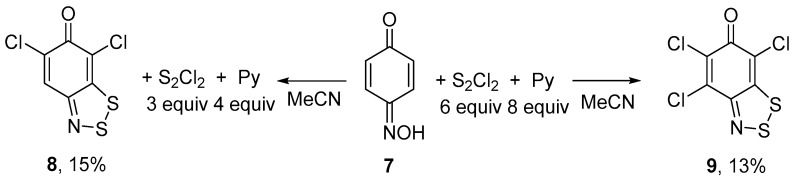
Reaction of oxime **7** with S_2_Cl_2_/pyridine to give dithiazoles **8** and **9**.

In the previous study on six-membered oximes, *p*-benzoquinone monooxime **7** gave a complex mixture of products on reaction with S_2_Cl_2_ and the desired fused dithiazole was obtained in a low yield [13]. We have found that the treatment of **7** with S_2_Cl_2_/pyridine in MeCN leads selectively to dichloro dithiazole **8** or to trichloro dithiazole **9** depending on the molar excess of the reagents. In boiling MeCN, reaction with a larger excess (S_2_Cl_2_, 6 equiv; pyridine, 8 equiv) gave **9**, whereas with a smaller excess (S_2_Cl_2_, 3 equiv; pyridine, 4 equiv) **8**, in both cases, however, in low yields (Scheme 4).

**Scheme 5:**
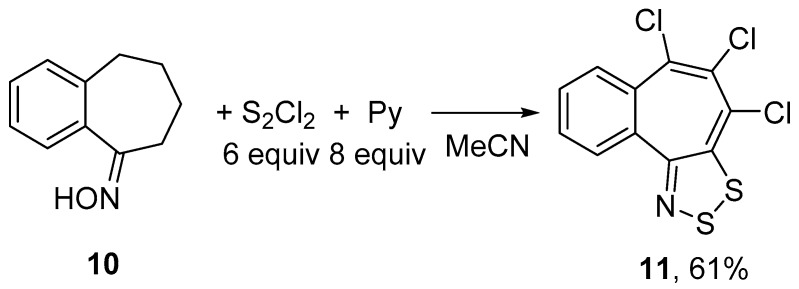
Reaction of oxime **10** with S_2_Cl_2_/pyridine to give dithiazole **11**.

Treatment of benzosuberone oxime **10** with S_2_Cl_2_/pyridine gave selectively 4,5,6-trichlorobenzo[6,7]cyclohepta[1,2-*d*][1,2,3]dithiazole **11** independent of the quantities of reagents; the best yield of **11** (61%) was obtained when 6 equiv of S_2_Cl_2_ and 8 equiv of pyridine were employed (Scheme 5).

**Scheme 6:**
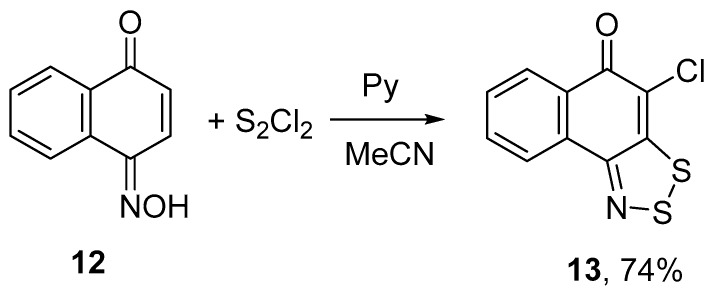
Reaction of oxime **12** with S_2_Cl_2_/pyridine to give dithiazole **13**.

**Figure 1:**
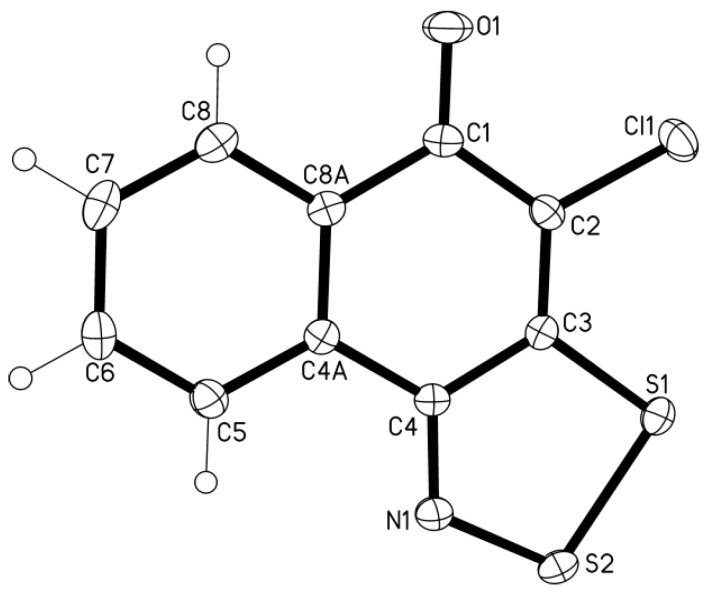
XRD molecular structure of dithiazole **13** (displacement ellipsoids at 50%, atoms H are shown as cycles). Selected bond distances (Å) and angles (^o^) (crystallographic numbering): C3-S1 1.7264(5), S1-S2 2.0774(5), S2-N1 1.6297(13), N1-C4 1.3054(19), C4-C3 1.456(2), C1-O1 1.2301(19), C2-Cl1 1.7259(16); C3-S1-S2 92.18(5), S1-S2-N1 98.13(5), S2-N1-C4 117.09(11), N1-C4-C3 118.65(13), C4-C3-S1 113.94(10).

1,4-Naphthoquinone oxime **12** treated with 3 equiv of S_2_Cl_2_ and 4 equiv of pyridine gave 4-chloro-5*H*-naphtho[1,2-*d*][1,2,3]dithiazol-5-one **13** in 74% yield (Scheme 6). The structure of **13** was confirmed by single-crystal XRD (Figure 1). 

The molecule is perfectly planar and the bond distances and angles correspond to the statistical means [20]. The crystal packing reveals shortened contacts S···O together with π-π and Cl-π interactions [21].

**Scheme 7:**
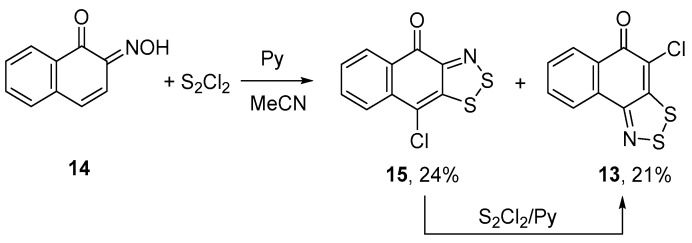
Reaction of oxime **14** with S_2_Cl_2_/pyridine to give dithiazoles **15** and **13**.

An unexpected result was obtained with naphthoquinone oxime **14**, an isomer of oxime **12**. Treatment of **14** with S_2_Cl_2_/pyridine in MeCN gave a mixture of two isomeric chloronaphthodithiazolones **13** and **15** in comparable yields (Scheme 7). First of all, it was shown that **14** was an individual compound with no traces of its isomer **12**. Special experiments on individual **13** and **15** showed that **15** converts into **13** when treated with S_2_Cl_2_/pyridine, whereas **13** remains unchanged. Effectively, the dithiazole ring moved from α- to β-position, with respect to the keto group, and the reaction under discussion represents a novel rearrangement. Earlier, similar processes were discovered by us for fused 1,2,3,4,5-pentathiepines [22,23,24]. Apparently, the mechanism of this rearrangement includes dithiazole ring-opening in **15** by the action of the chlorinating agent (*i.e.*, S_2_Cl_2_) followed by the ring-closure to afford **13** (Scheme 7). The scope of this rearrangement is under consideration.

### 2.2. Electrochemical Reduction and Oxidation of Dithiazole ***13***

Recently, it was shown that benzo-fused 1,2,3-dithiazoles are able to form persistent radical-anions (RAs) under conditions of electrochemical and chemical reduction and one of the RAs was isolated in the form of thermally-stable paramagnetic salts [6,7]. Amongst compounds synthesized in this work napthoquinone-fused derivatives **13** and **15** are especially interesting in the context of RAs since one may expect some concentration of a negative charge on the C=O moieties (ultimately leading to the C–O^−^ bonding situation) enlarging their ability to coordinate metal cations. This might be a new approach to the design and synthesis of sulfur-nitrogen π-heterocyclic RA salts as potential building blocks of magnetic functional materials [25,26,27,28,29].

**Figure 2:**
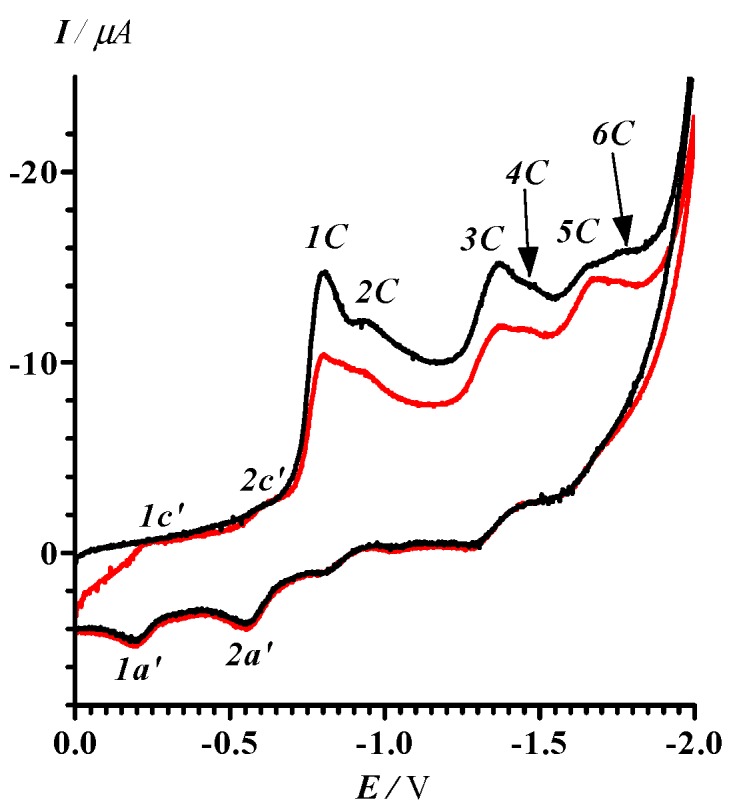
CV of **13** in MeCN (black curve: first cycle; red curve: second cycle). The potential sweep range was 0 > *E* > −2.0 V and the potential sweep rate 100 mV·s^–1^. Peak potentials (−$E_{p}^{ij}$ [30], V): 1C, 0.80; 2C, 0.93; 3C, 1.37; 4C, 1.47; 5C, 1.68, 6C, 1.76; 1c’, 0.24; 1a’, 0.19; 2c’, 0.62; 2a’, 0.56.

The electrochemical behavior of **13** was studied and found to be a very complex multistep process. Thus, the CV of **13** in MeCN (0 > *E* > −2.0 V) contains six irreversible peaks in the cathodic branch of the voltammogram. The additional quasi-reversible peaks 1c’−1a’ and 2c’−2a’ were observed in the second cycle at lower potentials than $E_{p}^{1C}$ (Figure 2).

**Figure 3:**
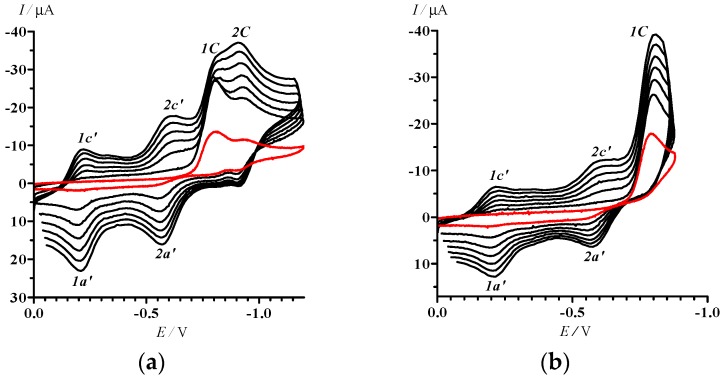
CVs of **13** in MeCN with potential sweep ranges 0 > *E* > −1.2 (**a**) and 0 > *E* > −0.88; (**b**) V. Potential sweep rates were 50 (first cycle, red curve), 250, 450, 650. 850, 1050, and 1250 (2–7 cycles, black curves) mV·s^–1^.

With a limited potential sweep covering only reduction peaks 1C and 2C (0 > *E* > −1.2 V), peaks 1c’–1a’ and 2c’–2a’ did not vanish (Figure 3a). Moreover, further decrease in the potential sweep down to the range 0 > *E* > −0.88 V embracing only the first irreversible step of the reduction of **13** (Figure 3b) did not cause any qualitative change in the compound’s CV. We conclude that both electrode processes 1c’–1a’ and 2c’–2a’ belong to the product(s) of transformation of **13** at the first step of its reduction, and their currents are kinetically controlled by the reactions at the peak 1C.

The electrochemical reduction of **13** could be accompanied by its rapid irreversible dechlorination initiated by electron transfer (*cf.* [31]). Additionally, the bond C=N of the 1,2,3-dithiazole ring can undergo irreversible reduction in the RA state of a molecule, or the ring can be opened by the cleavage of S-S or/and S-N bonds (*cf.* [32]). However, it is impossible to assign, unambiguously, these processes to the peaks 1C or 2C. No long-lived paramagnetic products were observed by conventional EPR spectroscopy under stationary electrolysis of **13** in the range of potentials −0.8 > *E* > −1.8 V.

**Figure 4:**
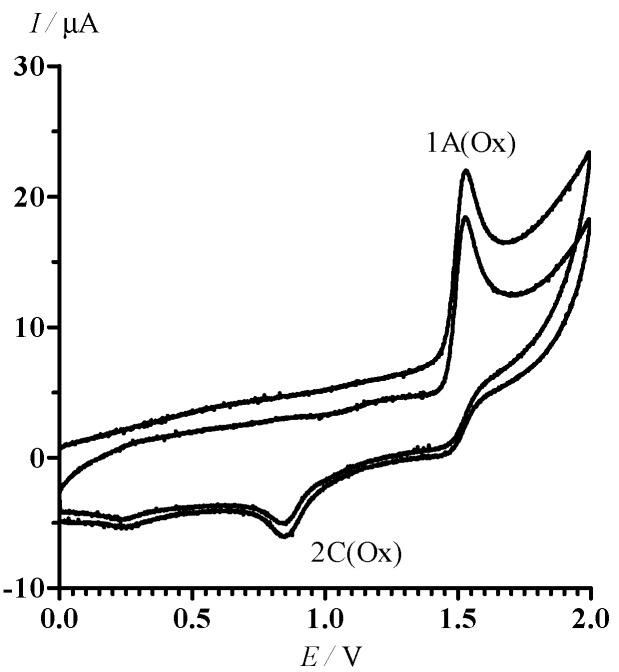
CV of **13** in MeCN in the range of potential sweep 0 < *E* < 2.0 V. The potential sweep rate was 100 mV·s^−1^.

CV of **13** in oxidative area of potentials is characterized by the only irreversible peak 1A (Ox) at the potential $E_{p}^{\mathrm{1A(Ox)}}$ = 1.53 V (Figure 4). An additional irreversible peak 2C (Ox) ($E_{p}^{\mathrm{2A(Ox)}}$ = 0.85 V) corresponding to the reduction of oxidation products of **13** is observed in the cathode branch of the CV.

Overall, the electrochemical behavior of **13** is characterized by a large number of multistage irreversible processes and their interpretation is a real challenge. Due to this complexity, at the current state of research only a qualitative description of the electrochemical behavior of **13** is possible. For further work preparative electrochemical reduction of **13** at controlled potentials is planned to obtain samples of reduction products enable their conventional characterization, together with generation of RAs from chlorine-less 1,2,3-dithiazoles such as **4** and related derivatives.

## 3. Experimental Section

### 3.1. General Information

Elemental analyses for C, H, and N were performed with Perkin Elmer 2400 Elemental Analyser (Perkin Elmer, Waltham, MA, USA). Melting points were determined on a Boetius hot-stage apparatus and are uncorrected.

^1^H (300.1 MHz) and ^13^C (75.5 MHz) NMR spectra were taken for CDCl_3_ solutions with a Bruker AM-300 (Bruker AXS Handheld Inc., Kennewick, WA, USA).

MS spectra (EI, 70 eV) were obtained with a Finnigan MAT INCOS 50 (Hazlet, NJ, USA), and high-resolution MS spectra with a Bruker micrOTOF II (Bruker Daltonik Gmbh, Bremen, Germany) instruments using electrospray ionization. The measurements were operated in a positive ion mode (interface capillary voltage −4500 V) or in a negative ion mode (3200 V); mass range was from *m*/*z* 50 to *m*/*z* 3000 Da; external or internal calibration was done with Electrospray Calibrant Solution (Fluka). A syringe injection was used for solutions in MeCN, methanol, or water (flow rate 3 µL·min^−1^). Nitrogen was applied as a drying gas; interface temperature was 180 °C.

IR spectra were measured with a Specord M-80 instrument (Carl Zeiss, Jena, Germany) in KBr pellets.

Oximes **3** [33], **7** [34], **12** [35] and **14** [36] were prepared according to the published procedures.

### 3.2. General Procedure for the Reaction of Cyclic Oximes with S_2_Cl_2_ and Pyridine in Acetonitrile

At −25 °C and under argon, S_2_Cl_2_ (0.24 mL, 3.0 mmol; or 0.48 mL, 6.0 mmol) was added dropwise to a stirred solution of oxime (1.0 mmol) and pyridine (0.32 mL, 4.0 mmol; or 0.64 mL, 8.0 mmol) in dry MeCN (10 mL). The mixture was stirred for 0.5 h at −5–0 °C, for 24 h at ambient temperature, refluxed for 1 h, filtered and the solvent was distilled off under reduced pressure. The residue was dissolved in EtOH (20 mL), diluted by H_2_O (20 mL) and extracted with ether (3 × 20 mL). Combined extracts were washed with H_2_O (20 mL), dried, and solvent was evaporated.

*8-Chloroindeno[1,2-d]-1,2,3-dithiazole* **2**. Red solid, mp 108–110 °С (107–109 °С [12]). IR and MS spectra are similar to the literature data [12].

*8-Phenylindeno[1,2-d]-1,2,3-dithiazole* **4**. Yellow solid, mp 111–112 °С (111–113 °С [12]). IR and MS spectra are similar to the literature data [12].

*4,5,6-Trichlorocyclopenta[d][1,2,3]dithiazole* **6a**. Deep purple solid, mp 122–124 °С (125–127 °С [12]). IR and MS spectra are similar to the literature data [12].

*Ethyl 5,6-dichlorocyclopenta[d][1,2,3]dithiazole-4-carboxylate* **6b**. Yellow solid, mp 80–82 °С (83–84 °С [15]). IR and MS spectra are similar to the literature data [15].

*5,7-Dichloro-6H-1,2,3-benzodithiazol-6-one* **8**. Red solid, mp 259–261 °С (257–258 °С [13]). IR and MS spectra are similar to the literature data [13].

*4,5,7-Trichloro-6H-1,2,3-benzodithiazol-6-one* **9**. Red solid, mp 214–215 °С (216–217 °С [13]). IR and MS spectra are similar to the literature data [13].

*4,5,6-Trichlorobenzo[6,7]cyclohepta[1,2-d][1,2,3]dithiazole* **11**. Red solid, mp 119–121 °С (121–122 °С [12]). IR and MS spectra are similar to the literature data [12].

*4-Chloro-5H-naphtho[1,2-d][1,2,3]dithiazol-5-one* **13**. Red solid, mp 230–233 °С (234–235 °С [13]). IR and MS spectra are similar to the literature data [13]. 

*9-Chloro-4H-naphtho[2,3-d][1,2,3]dithiazol-4-one* **15**. Blue solid, mp 192–195 °С (195–196 °С [13]). IR and MS spectra are similar to the literature data [13].

All compounds synthesized had correct elemental analyses and NMR spectra.

### 3.3. Behavior of 1,2,3-Dithiazoles ***13*** and ***15*** in the S_2_Cl_2_/Pyridine System

At −25 °C and under argon, S_2_Cl_2_ (0.048 mL, 0.6 mmol) was added dropwise to a stirred solution of **15** (25 mg, 0.1 mmol) and pyridine (0.064 mL, 8.0 mmol) in dry MeCN (3 mL). The mixture was refluxed for 2 h, filtered and the solvent was distilled off under reduced pressure. The residue was dissolved in CH_2_Cl_2_ (7 mL), washed with H_2_O (3 × 2 mL), dried over MgSO_4_, and the residue was separated by flash chromatography (silica gel Merck 60, hexane to hexane/CH_2_Cl_2_ mixtures) to give **13** (10 mg, 39%) and **15** (11 mg, 43%).

In the experiment with **13** under the same reaction conditions it was quantitatively recovered.

### 3.4. X-ray Diffraction

XRD data of **13** were obtained with a Bruker Kappa Apex II CCD diffractometer (Bruker AXS Gmbh, Karlsruhe, Germany) using φ, ω scans of narrow (0.5°) frames with Mo Kα radiation (λ = 0.71073 Å) and a graphite monochromator. The structure of **13** was solved by direct methods and refined by full-matrix least-squares method against all *F*^2^ in anisotropic approximation using the *SHELX-97* (Bruker AXS, Madison, WI, USA) programs set [37]. The H atoms positions were calculated with the riding model. Absorption corrections were applied empirically using *SADABS* programs [38]. Shortened intermolecular contacts were analyzed using the *PLATON* [39] and *MERCURY* [40] programs.

Compound **13** is orthorhombic, space group *Pca*2_1_, *a* = 16.6153(6), *b* = 3.8775(1), *c* = 15.4168(6) Å, *V* = 993.24(6) Å^3^, *Z* = 4, C_10_H_4_ClNOS_2_, *D_calc_* = 1.697 g·cm^–3^, μ = 0.770 mm^–1^, F(000) = 512, crystal size 0.80 × 0.20 × 0.07 mm^3^, independent reflections 2254 (R_int._ = 0.0404), wR_2_ = 0.0512, S = 1.09 for all reflections (R = 0.0196 for 2202 F > 4σ). Tables listing detailed crystallographic data, atomic positional parameters, and bond lengths and angles are available as CCDC 1468963 from the Cambridge Crystallographic Data Centre via www.ccdc.cam.ac.uk/data_request/cif.

### 3.5. Cyclic Voltammetry

The CV measurements on compound **13** (~1.2 mM solutions in MeCN) were performed with a PG 310 USB potentiostat (HEKA Elektronik, Germany) at 293 K in an argon atmosphere at a stationary Pt spherical electrode (S = 0.08 cm^2^) with 0.1 M Et_4_NClO_4_ as a supporting electrolyte. A standard electrochemical cell (solution volume was 5 mL) connected to the potentiostat with three-electrode scheme was used. Peak potentials were quoted with reference to a saturated calomel electrode (sce).

## 4. Conclusions

In this work, a new general procedure for the selective synthesis of carbocycle-fused 1,2,3-dithiazoles based on the reaction of cyclic oximes with S_2_Cl_2_ and pyridine in MeCN was established. With naphthalenone derivatives, a novel dithiazole rearrangement was discovered, *i.e.*, isomerization of **15** into **13**. The structure of 1,2,3-dithiazole **13** was confirmed by single-crystal XRD. 

In most cases the hetero ring-closure was accompanied by chlorination. The presence of chlorine atoms in the 1,2,3-dithiazoles synthesized most likely causes instability of their reduced forms. Particularly, under the CV conditions and with compound **13**, we speculate that hydrodechlorination occurs at a low potential. In any case, no long-lived paramagnetic products were observed by conventional EPR spectroscopy under stationary electrolysis of **13** in the potential range −0.8 > *E* > −1.8 V. In further syntheses of carbocycle-fused 1,2,3-dithiazoles as potential precursors of persistent RAs (*cf.* [6,7]) using the established procedure the chlorination must be prevented by appropriate substitution in starting oximes.

The effectiveness of the S_2_Cl_2_/pyridine system in the chemistry described in this work motivates a special investigation of possible interaction/complexation between its components.
